# Correlated Evolution of Nucleotide Positions within Splice Sites in Mammals

**DOI:** 10.1371/journal.pone.0144388

**Published:** 2015-12-07

**Authors:** Stepan Denisov, Georgii Bazykin, Alexander Favorov, Andrey Mironov, Mikhail Gelfand

**Affiliations:** 1 A. A. Kharkevich Insitute for Information Transmission Problems RAS, Moscow, Russia; 2 Faculty of Bioengineering and Bioinformatics, M. V. Lomonosov Moscow State University, Moscow, Russia; 3 Division of Oncology Biostatistics, The Sidney Kimmel Comprehensive Cancer Center at Johns Hopkins, Baltimore, Maryland, United States of America; 4 Laboratory of System Biology and Computational Genetics, Department of Computational System Biology, N. I. Vavilov Institute of General Genetics, Moscow, Russia; 5 Laboratory of Bioinformatics, State Research Institute of Genetics and Selection of Industrial Microorganism (GosNIIGenetika), Moscow, Russia; International Centre for Genetic Engineering and Biotechnology, ITALY

## Abstract

Splice sites (SSs)—short nucleotide sequences flanking introns—are under selection for spliceosome binding, and adhere to consensus sequences. However, non-consensus nucleotides, many of which probably reduce SS performance, are frequent. Little is known about the mechanisms maintaining such apparently suboptimal SSs. Here, we study the correlations between strengths of nucleotides occupying different positions of the same SS. Such correlations may arise due to epistatic interactions between positions (i.e., a situation when the fitness effect of a nucleotide in one position depends on the nucleotide in another position), their evolutionary history, or to other reasons. Within both the intronic and the exonic parts of donor SSs, nucleotides that increase (decrease) SS strength tend to co-occur with other nucleotides increasing (respectively, decreasing) it, consistent with positive epistasis. Between the intronic and exonic parts of donor SSs, the correlations of nucleotide strengths tend to be negative, consistent with negative epistasis. In the course of evolution, substitutions at a donor SS tend to decrease the strength of its exonic part, and either increase or do not change the strength of its intronic part. In acceptor SSs, the situation is more complicated; the correlations between adjacent positions appear to be driven mainly by avoidance of the AG dinucleotide which may cause aberrant splicing. In summary, both the content and the evolution of SSs is shaped by a complex network of interdependences between adjacent nucleotides that respond to a range of sometimes conflicting selective constraints.

## Introduction

Splicing is an important step of gene expression in eukaryotes. Alternative splicing generates multiple RNA isoforms from one transcript, allowing one gene to produce different proteins in a tissue-specific and time-specific manner. In eukaryotes, splicing requires a spliceosome, an RNA-protein complex comprised of five snRNAs (U1, U2, U4, U5 and U6) and about 200 proteins that are conserved in eukaryotes [1]. For an intron to be spliced, it requires a pair of functional splice sites (SSs), sequences of several nucleotides at the exon-intron boundaries that directly interact with the spliceosome. The donor splice site (DSS) is located at the 5’ end of an intron, and the acceptor splice site (ASS) is located at the 3’ end of an intron. Both DSSs and ASSs have intronic and exonic parts. The intronic parts are longer than the exonic ones. SSs with different nucleotide sequences may have different degrees of affinity to a spliceosome. The consensus SS sequences are the same in all eukaryotes: (A/C)**AG**|GT(A/G)AGT for the DSSs, and (T/C)_n_NC**AG|**G for the ASSs; here, vertical lines correspond to the exon-intron boundary, and (T/C)_n_ is the polypyrimidine tract (PPT). With the exception of some minor classes of SSs (U12-sites, U2-type GC-AG and a few others [2]), all SSs have an invariant dinucleotide: GT for DSSs, and AG for ASSs (shown in boldface in the consensus sequences above) [3]. The branch point, which is located upstream of the ASS within an intron, is also important for splicing, but is not considered here, as its sequence is degenerate, and have been experimentally defined only for about 20% of introns [4,5]. Individual nucleotides of SSs form base pairs with specific positions of snRNAs, or are involved in binding spliceosomal proteins (protein binding is not necessarily specific, especially in the PPT). Components of the spliceosome involved in such interactions are overall highly conserved in eukaryotes, implying that these interactions should also be similar in different species. Nevertheless, individual components of this machinery may be dispensable.

Specifically, in the DSSs, the 5’ end of the U1 snRNA binds directly to the SS [6,7], and one of the important factors influencing the DSS functionality is its complementarity to the U1 snRNA [8]. U1 can base-pair with positions -3 to +6 of the DSS [9]; those U1 nucleotides that bind to positions -2 to +5 are absolutely conserved throughout eukaryotes [10,11]. The nucleotide G of U1, which pairs with -3(C), is conserved in animals [11]. The first step in the spliceosome assembly, the formation of the E-complex, depends on the base pairing between U1 and the DSS [12–14]. Further in the splicing process, U1 is replaced by U5 and U6 snRNAs. U5 can base-pair with positions -3 to +1, and U6 can base-pair with positions +5 and +6 of the DSS [9]; however, the base pairing of the invariant loop of U5 with the exonic positions is dispensable for splicing in the HeLa nuclear extract [15].

In the ASSs, specific proteins bind to the SS at the stage of the E-complex formation. U2AF65 interacts with the PPT, U2AF35 interacts with the AG dinucleotide, and SF1 binds to the branch point and facilitates binding of U2AF65 to the adjacent PPT [16,17]. All metazoans have functional homologs of all three of these proteins. RNA-recognition motifs RRM1 and RRM2 of U2AF65, which bind to PPT, are absolutely conserved in the human, mouse and dog genomes [11].Binding of U2AF65 promotes U2 snRNA-branch point base pairing[18]. It is not clear which of the two snRNPs—U1 or U2—comes first [19]. Both scenarios yield prespliceosomes assembly, followed by U5·U4/U6 tri-snRNP recruitment and intron excision.

Because the GT and AG dinucleotides are necessary for splicing, they are almost invariably present at intron termini, and very well conserved between species (e.g. [20]). Conversely, other SS positions vary to some extent, both between different SSs within a genome and between orthologous SSs in different species, and are not necessarily occupied by the consensus nucleotides.

The degree of adherence of a SS position to the consensus can be characterized by its strength; the strength of an entire SS can be then defined as the sum of the strengths of its positions. Notably, SSs of alternative exons tend to have, on average, lower strength than constitutive SSs [21–25]. In some cases, selection may favor weaker SSs over stronger ones [25]. Several experimental studies showed that strengthening of weak alternative SSs may cause loss of splicing regulation [9,26]. However, at least in SSs of constitutive exons, there is no specific selection for SSs to be weak: more likely, weak SSs are preferred because of other competing selective constraints probably not connected to splicing itself [27].

SS strength depends on the age of the corresponding intron [28]. Sverdlov et al. classified introns into ‘old’ introns conserved in two or more major eukaryotic lineages, and ‘young’ lineage-specific introns. DSSs of old introns have stronger exonic parts, and weaker intronic parts, compared to DSSs of new introns. In ASSs of old introns, the exonic part is also stronger than in ASSs of young introns, although no difference was observed between intronic parts of ASSs of different ages [28]. These results are in agreement with the intron-late hypothesis of intron origin [29]. According to this hypothesis, the ancestor of all eukaryotic organisms had intron-free genes; in the course of subsequent eukaryotic evolution, introns were inserted into so-called protosplice sites, i.e. sequences in coding regions that happened to be similar to the consensus sequences of exonic parts of SSs [30,31]. For this to happen, the original proto-splice site had to be strong; during subsequent evolution, the signal migrated from the exonic to the intronic part of the SS, causing strengthening of the intronic and weakening of the exonic signal.

This migration is caused by a conflict between selection pressures on splicing efficiency and protein sequence [32]. Old splice sites show how this conflict is ultimately resolved: they have comparatively strong intronic parts (to splice efficiently), and comparatively weak exonic parts (not to interfere with the requirements of coding a specific protein). Sverdlov et al. addressed the nature of the hypothesized proto-splice sites by analyzing the sequence of exonic junctions overlapping a codon that encoded an amino acid invariant in all eukaryotes [33]. They suggested that such amino acids were likely inherited from the common eukaryotic ancestor and, consequently, represent “frozen” protosplice sites. It was shown that such protosplice sites have the consensus sequence (A/C)AG||GT (the position of the intron is shown by the two vertical lines), thus matching the consensus sequence of contemporary sites. This implies that the positions of protosplice sites were not random with regard to the coding sequence where they originated; and that their exonic parts were initially strong.

Although the key components of the spliceosome are conserved in all eukaryotes, the average strength of SSs varies between large eukaryotic groups. Intron-poor organisms (e.g. budding yeast *Saccharomyces cerevisiae*) have stronger SSs than intron-rich ones (e.g. vertebrates) [34]. However, there is little, if any, difference in SS profiles, i.e. position-specific nucleotide frequencies, between more closely related species. Specifically, the differences between the human and mouse DSSs profiles are negligible [9].

Despite this high conservation of species-specific SS profiles, individual nucleotides within SSs can evolve between species. Here, we study this evolution in the genomes of three mammals at moderate phylogenetic distances from each other: human, mouse, and dog. We ask how selection affects the evolution of nucleotides at different SS positions, and specifically, how it shapes the adherence to the consensus.

The first question we ask is whether SSs tend to evolve towards the consensus. Here, we systematically analyze substitutions at SS positions in human, mouse, and dog lineages since their last common ancestor, and ask whether they led to an increase or a decrease of the SS strength. In particular, we tested one prediction of the signal-migration hypothesis, namely that the exonic parts of SSs decrease their strengths and intronic parts increase their strengths in the course of evolution.

The second question we ask is whether adherence to consensus in different SS positions is correlated. Several previous studies have shown that nucleotides of different positions within SSs are not independent. G at position -1 and A at position -2 of DSSs occur rarely if position +5 is occupied by G [35]. Frequency of A at position +4 and T at position +6 is greater if position +6 is occupied by G [35]. Statistical associations were noticed between positions +5 and -1, +5 and -2, +5 and -3, -2 and -1, +3 and +4, +5 and +6 in DSSs; and between positions -3 and +1, -7 and -6, -6 and -5 in ASSs [36]. A comparative analysis of orthologous human and mouse DSSs have shown than a number of changes at the pairs of positions -1 and +5, and -2 and +5, was lower than expected if these positions had evolved independently, while at pairs of positions +4 and +5, and +5 and +6, it was higher than expected [37].

Here, we reformulate the question of interactions between positions in terms of correlations between position strengths and analyze it systematically. We confirm previous work, and detect novel correlations. Moreover, we analyze what evolutionary forces have led to the existence of the observed correlations.

A correlation between SS positions in their adherence to the consensus can arise due to several reasons, including epistatic selection and SS history. Epistasis is a phenomenon whereby the contribution to organismal fitness of a particular allele at one locus depends on the allele at another locus. We consider positions within a SS as loci, and nucleotides occupying these positions, as alleles. Epistatic selection, when the strength and/or direction of selection at one position depends on the allele at another position, can lead to non-independence of SS composition and evolution between positions. On the other hand, such a non-independence may arise due to the evolutionary history of SSs. SSs of new introns have stronger exonic parts and weaker intronic parts than those of old introns [28]. Pooling of introns of different ages can lead to correlations between strengths of positions within a SS.

## Materials and Methods

### Sample

SSs were extracted from the EDAS database (http://edas2.bioinf.fbb.msu.ru/, [38]); only sites with canonical AG (for DSSs) and GT (for ASSs) dinucleotides were used. Genomic coordinates of orthologous DSSs and ASSs from the *Homo sapiens*, *Mus musculus* and *Canis familiaris* genomes were obtained as described previously [39,40]. Based on these coordinates, we extracted, from each of the three genomes, the sequences of each SS and its genomic neighborhood, including the canonical dinucleotide of the SS, 18 adjacent intronic nucleotides, and 10 adjacent exonic nucleotides, for a total of 30 nucleotides.

We considered only those SSs that bound cassette and constitutive exons, defined by the human transcriptomics data. Specifically, an exon was considered constitutive if (i) it was supported by at least 50 human transcripts (ESTs or mRNAs), and (ii) it was skipped in fewer than 5% of sequences that covered the corresponding region, i.e. the exon inclusion frequency was above 95%. A cassette exon was considered if it was included in between 5% and 95% of all sequences that covered the corresponding region (no limit was set for the number of supporting transcripts). Exons not matching either of these definitions were excluded from analyses. We considered exons only within coding regions of genes; a cassette exon was allowed to contain a stop codon only if skipping of this exon extended the ORF. A total of 12,245 triples of DSSs and 12,245 triples of ASSs were considered at this stage.

To test the robustness of our analyses to changes of transcription patterns between species, we constructed two datasets of SSs: (i) using only the human transcriptomics data as described above, and (ii) additionally requiring, for each SS, an observation of splicing consistent with this SS in at least one mouse EST. The reported results were obtained using the second dataset. This filtering reduced the sample to 4,388 DSSs and 4,411 ASSs, including 3,596 constitutive DSSs, 791 cassette DSSs, 3,601 constitutive ASSs, and 810 cassette ASSs (S1 Dataset). Our filtering biased the distribution of exon inclusion rates of cassette-exon SSs very slightly towards low values (S1 Table).

Constitutive DSSs were subdivided into *young* and *old* according to multispecies conservation of the key dinucleotide (AG or GT) as described in [27]. DSSs with phylogenetic distances from *Homo sapiens* genome < 1 K_s_ unit (i.e., placental mammals) were considered young, and with ≥ 1K_s_ units, old. This procedure resulted in 1,655 young and 1,923 old constitutive DSSs (S1 Dataset). 19 DSSs were discarded because they were not uniquely mapped to the human genome.

### Estimation of the transition matrix

**Fig 1:**
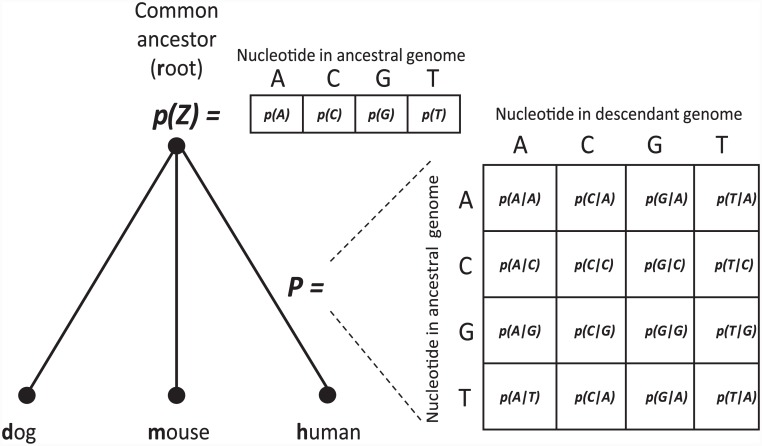
Substitution matrix and vector of ancestral probabilities. We assume a simplified phylogenetic tree in which the branches leading to the extant organisms (human, mouse and dog) trifurcated from their last common ancestor. For each position within a SS, a separate substitution matrix *P* was constructed for each of the three phylogenetic branches (only the human substitution matrix is shown for simplicity). Rows in *P* correspond to nucleotides in the ancestral genome, and columns, to nucleotides in the descendant genome (here, human). An element of *P* is the conditional probability *p(X|Z)* that a nucleotide *Z* at this position in the ancestral genome was replaced with nucleotide X in the descendant genome. Elements of the vector of ancestral probabilities *p(Z)* are probabilities of each nucleotide in the ancestral genome.

The internal branch connecting the common ancestor of *H*. *sapiens* and *M*. *musculus*, and the common ancestor of *H*. *sapiens*, *M*. *musculus*, and *C*. *familiaris*, is relatively short (e.g. [41]). Thus, to simplify the model, we assumed that the branches leading to the extant organisms (human, mouse, and dog) trifurcated at the common ancestor, referred to as the ancestral genome (Fig 1).

To analyze the evolutionary process, we assigned, to each of the three tree branches and to each nucleotide position within a SS, a 4×4 nucleotide substitution matrix *P*. An element of this matrix is the conditional probability *p(X|Z)* that a nucleotide *Z* at the given position in the ancestral genome was substituted to a nucleotide X in the descendant genome. This description also includes the vector of ancestral nucleotide probabilities *p(Z) = (p(Z = A)*, *p(Z = C)*, *p(Z = G)*, *p(Z = T))*. For example, if we know the number of substitutions, say, from A to G between the ancestor and the human #(A→G) and the number of A’s in the ancestor *#A* (and also *#C*, *#G and #T*), then a good estimate of *p(G|A)* would be *#(A→G)/ #A*, and a good estimate of *p(Z = A) would be #A/(#A + #C + #G + #T)*. However, we don’t know the numbers of substitutions. Therefore, we estimate *p(Z)* and *P* from data as follows.

We estimated *p(Z)* for each nucleotide position of a SS, and *P* for each branch of the evolutionary tree at this position, implementing the parameter-rich model of nucleotide substitutions [42]. For each triple SS alignment, we denoted, for each position of the SS, the nucleotide in the ancestor, human, mouse, and dog, as *r*, *h*, *m*, and *d*, respectively. Next, we pooled together all position-specific triples *(hmd)* from all SS alignments, and, for each of the 64 possible triples (AAA, AAC, …, TTT), calculated its frequency *q(AAA)*, *q(AAC)*, *…*, *q(TTT)*. The method is based on minimization of the difference between the model-estimated probabilities of triples *p(AAA)*, *p(AAC)*,*…*, *p(TTT)* and the observed frequencies of triples. The model estimates the probabilities of triples as follows:

$$\begin{array}{c} p(AAA)=\sum_{r}p(r)p(h=A|r)p(m=A|r)p(d=A|r)\\ p(AAC)=\sum_{r}p(r)p(h=A|r)p(m=A|r)p(d=C|r)\\ \ldots \\ p(TTT)=\sum_{r}p(r)p(h=T|r)p(m=T|r)p(d=T|r) \end{array}$$ (1)

We obtain a system of 64 equations with 39 independent variables (three matrices of sixteen variables with four ties each, and the ancestral vector of four variables with one tie). An approximate solution of this overdetermined system of equations was obtained by the least squares method using MATLAB function fmincon() (http://www.mathworks.com/help/optim/ug/fmincon.html). We minimized the function *f(P*, *p(Z)) = (p(AAA)-q(AAA))* ^*2*^ *+ (p(AAC)-q(AAC))* ^*2*^ *+ … + (p(TTT)-q(TTT))* ^*2*^. We started from random initial conditions 10 times, and retained the lowest of the observed local minima. In most cases, all 10 iterations resulted in the same minimum.

In addition, we compared our likelihood-based (LB) method of transition matrix estimation with the standard parsimony-based method (PB). Since PB minimizes the number of substitutions, in all estimated transition matrices, the probability of a nucleotide to be conserved between the ancestral and a descendant SS position was almost always greater in PB than in LB.

### Estimation of the ancestral site probabilities

Assuming that different positions in ancestral SSs are mutually independent, the probability of an ancestral SS *R* (root) given descendant SSs *H*, *M*, and *D* is

$$p(R|HMD)=\prod_{i=1}^{L}p(r_{i}|h_{i}m_{i}d_{i}),$$

where *p*(*r*_*i*_|*h*_*i*_ *m*_*i*_ *d*_*i*_) is the probability of nucleotide *r*_*i*_ at position *i*, given descendant nucleotides *h*_*i*_, *m*_*i*_ and *d*_*i*_.

Since all branches evolve independently,

$$p(r_{i}h_{i}m_{i}d_{i})=p(r_{i})p(h_{i}|r_{i})p(m_{i}|r_{i})p(d_{i}|r_{i})$$

By the Bayes theorem:

$$p(r_{i}|h_{i}m_{i}d_{i})=\frac{p(r_{i}h_{i}m_{i}d_{i})}{\sum_{x\in \{A,C,G,T\}}p(xh_{i}m_{i}d_{i})}=\frac{p(r_{i})p(h_{i}|r_{i})p(m_{i}|r_{i})p(d_{i}|r_{i})}{\sum_{x\in \{A,C,G,T\}}p(x)p(h_{i}|x)p(m_{i}|x)p(d_{i}|x)}$$ (2)

Therefore,

$$p(R|HMD)=\prod_{i=1}^{L}\frac{p(r_{i})p(h_{i}|r_{i})p(m_{i}|r_{i})p(d_{i}|r_{i})}{\sum_{x\in \{A,C,G,T\}}p(x)p(h_{i}|x)p(m_{i}|x)p(d_{i}|x)}$$ (3)

### SS strength

SS strength (site weight, site score, see [22]) is a measure of its closeness to the consensus. Here, we consider only the strength *S(i)* of a single SS position *i*, which is defined as

$$W(S(i))=\text{log}\frac{p(S(i)|F)}{p(S(i)|B)}=\text{log}\frac{p_{F}(S(i),i)}{p_{B}(S(i),i)}$$ (4)

where *p(S(i)|F)* is the probability of the nucleotide *S(i)* assuming that *S(i)* is the *i*-th position of SS of the considered type (the foreground model); *p(S(i)|B)* is the probability of the nucleotide *S(i)* given that it is not in a SS (the background model); *p*_*F*_ *(α*, *i)* is the probability of the nucleotide *α* at position *i* in the foreground model, calculated from the statistics of the nucleotide occurrence for a given type of SS collected in a position weight matrix (PWM); *p*_*B*_ *(α*, *i)* is the probability of the nucleotide *α* at position *i* in the background model.

To account for the possibility that the observations depend on the selection of SSs for the PWM construction, we calculated strengths of SS positions using both the PWM based on ancestral sites and the PWM based on all descendant sites (human, mouse, and dog combined). The results were very similar; the presented results were obtained using the latter approach.

We used the simplest background model: *p*_*B*_ *(α*, *i) = 0*.*25* for each nucleotide *α* and each position *i*. As we are interested not in the SS strengths per se, but in the difference of strengths, the choice of the background model has no effect on the results.

If we know the frequencies *q(α*,*i)* of all nucleotides *α* in all positions *i* in a sample of SSs, we can calculate the average strength of *i*-th position of SSs in the sample as

$$\hat{W}(i)=\sum_{\alpha }q(\alpha ,i)\text{log}\frac{p_{F}(\alpha ,i)}{p_{B}(\alpha ,i)}$$ (5)

The expectation of an ancestral strength of position *i* given descendant SS positions *h*_i_, *m*_*i*_ and *d*_*i*_ in human, mouse and dog genomes respectively is

$$\bar{W}(i)=\sum_{r_{i}}W(r_{i})p(r_{i}|h_{i}m_{i}d_{i})$$ (6)

where *r*_*i*_ spans all possible nucleotides.

### Testing the hypothesis of independent evolution of distinct positions

To investigate the possible influence of the dependence between positions on SS evolution, we performed computational modeling. In this section, we consider human SSs as an example; for mouse and dog, the calculations were similar. We compared two samples of SSs: 1) real SSs observed in the human genome, and 2) artificial SSs which were observed in the simulation under the assumption that positions evolve independently. Artificial SSs were simulated as follows.

Consider any given triple of descendant sites *HMD* and all possible ancestral sites *{R}*. Assuming independent evolution, the probability of a simulated descendant site H* in the human genome given real sites *H*, *M* and *D* in the human, mouse and dog genomes, respectively, is

$$p(H^{*}|HMD)=\prod_{i=1}^{L}p({h_{i}}^{*}|h_{i}m_{i}d_{i})$$

where

$$p({h_{i}}^{*}|h_{i}m_{i}d_{i})=\sum_{r_{i}\in \{A,C,G,T\}}p({h_{i}}^{*}|r_{i},h_{i}m_{i}d_{i})p(r_{i}|h_{i}m_{i}d_{i})$$

The probability of h* depends directly only on the ancestor *r*_*i*_, *p*(*h*_i_*|*r*_*i*,_ *h*_*i*_ *m*_*i*_ *d*_*i*_) = *p*(*h*_l_*|*r*_*l*_), and is calculated using the same transition matrix as *p*(*h*_*i*_|*r*_*i*_).

From (Eq 2), we obtain

$$p({h_{i}}^{*}|h_{i}m_{i}d_{i})=\sum_{r_{i}\in \{A,C,G,T\}}\frac{p({h_{i}}^{*}|r_{i})p(r_{i})p(h_{i}|r_{i})p(m_{i}|r_{i})p(d_{i}|r_{i})}{\sum_{x\in \{A,C,G,T\}}p(x)p(h_{i}|x)p(m_{i}|x)p(d_{i}|x)}$$ (7)

We performed Monte-Carlo simulations using (Eq 7), obtaining a triple of simulated sites H*M*D* for each triple *HMD* from the sample. We then obtained strength vectors, i.e., vectors whose elements are the strengths of each SS position, for real and artificial SSs; and compared the covariance matrices of these vectors. The covariance matrix of strength vectors for all pairs of positions calculated for a sample of sites (e.g. all real sites in the human genome *{H}*) shows the dependence between strengths of positions. Negative values mean that two positions are anticorrelated (a nucleotide of a large strength in one position frequently co-occurs with a nucleotide of a low strength in the other position and vice versa). Positive values mean that low (large) strengths in the first position tends to co-occur with low (respectively, large) strengths in the second position. The comparison of the covariance matrices of real and simulated sites of the same organism allows us to infer whether evolutionary dependence between positions results in strengthening or weakening of the covariance, or has no effect.

### Neutral controls

For each position *i* of a site, we define the neutral transition matrix *P*_*E*_ *(i)* expected under the assumption of no specific selective pressure at this position (as opposed to the observed, actual transition matrix *P*_*O*_ *(i)*). To construct neutral matrices, we used intronic and exonic regions adjacent to SSs. We calculated transition matrices (and joint probability matrices) for these positions using the same algorithm as for the SS positions. In order to obtain the expected matrix, we summed the joint probability matrices from this region and then normalized them.

We used different quasi-neutral regions for different site positions. For the intronic part of the DSS, we used the adjacent intronic region (positions +7…+12). This guarantees exclusion of the SS sequence from the control, and also minimizes the probability of inclusion of any unannotated exons, since such exons are unlikely to be located this close to the DSS. Since the PPT of a ASS is a relatively long sequence with poorly defined boundary, we cannot use as a neutral control the intronic positions near the ASS. Instead, we used as a neutral control the intronic region of the DSS of the same exon.

The exonic positions of the DSS and ASS have frame-specific expected matrices. For the DSS, we used positions -4 and -7 (-5 and -8; -6 and -9) for the construction of the expected matrix for position -1 (respectively, -2; -3). Similarly, positions +4 and +7 (+5 and +8; +6 and +9) were used as a neutral control for positions +1 (respectively,+2; +3) of the ASS.

If for a given position *i* we know the ancestral vector of nucleotide probabilities *p(ancestor*, *i)* and the expected matrix *P*_*E*_ *(i)*, we can calculate the expected descendant vector of nucleotide probabilities *p*_*E*_ *(descendant*, *i) = P*_*E*_ *(i)×p(ancestor*, *i)*. We can estimate the observed descendant vector of nucleotide probabilities *p*_*O*_ *(descendant*, *i)* from the existing sample of SSs.

We define the expected descendant strength *Ŵ*_*E*_ *(descendant*, *i)* as the average strength of a position according to (Eq 5), where frequencies of nucleotides are obtained from vector *p*_*E*_ *(descendant*, *i)*. Analogously, we define the observed descendant strength *Ŵ*_*O*_ *(descendant*, *i)*. The ancestral strength is the average strength of a position with nucleotide frequencies obtained from *p(ancestor*, *i)*.

Thus, for each given position *i* we calculate the observed strength change *ΔŴ*_*O*_ *(i)* = *Ŵ*_*O*_ *(descendant*, *i)*—*Ŵ(ancestor*, *i)* and the expected strength change *ΔŴ*_*E*_ *(i)* = *Ŵ*_*E*_ *(descendant*, *i)*—*Ŵ(ancestor*, *i)*.

### Bootstrapping

The statistical significance of results was estimated by bootstrapping site triples in a total of 1,500 bootstrapping trials. One- and two-sample hypotheses testing and the calculation of the 95% confidence intervals for the statistics of interest were performed as described in [43].

### Control of dinucleotide content in ASSs

In order to understand whether the periodic pattern of correlations in region -12…-7 of ASSs is caused by dinucleotide content, we performed a simulation as follows. We generated a sample of artificial ASSs of the same size as the original ASS sample, keeping the region before position -12 and after position -7 the same as in the original ASSs, but replacing the region -12…-7 with artificially generated sequences. Artificial sequences in positions -12…-7 were generated using a first-order Markov chain with initial nucleotide probabilities estimated as nucleotide frequencies, and transition probabilities estimated from dinucleotide frequencies in this region. This control design preserves any dependence between adjacent positions, but disrupts more distant dependencies.

## Results

### Positions within a SS are not independent

**Fig 2:**
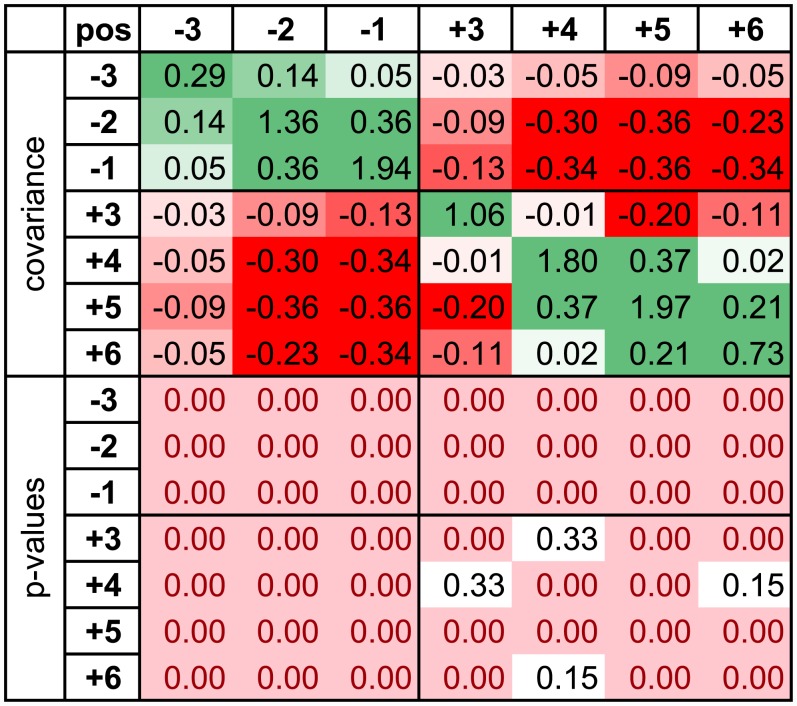
Covariance matrix of strength vectors for constitutive-exon DSSs (*M*. *musculus*). Top, covariance matrix, with positive covariance values in green, negative values in red, and color depth depending on the absolute value. Bottom, p-values that the given covariance is significantly different from zero obtained from bootstrapping; p-values < 0.05 are in pink.

**Fig 3:**
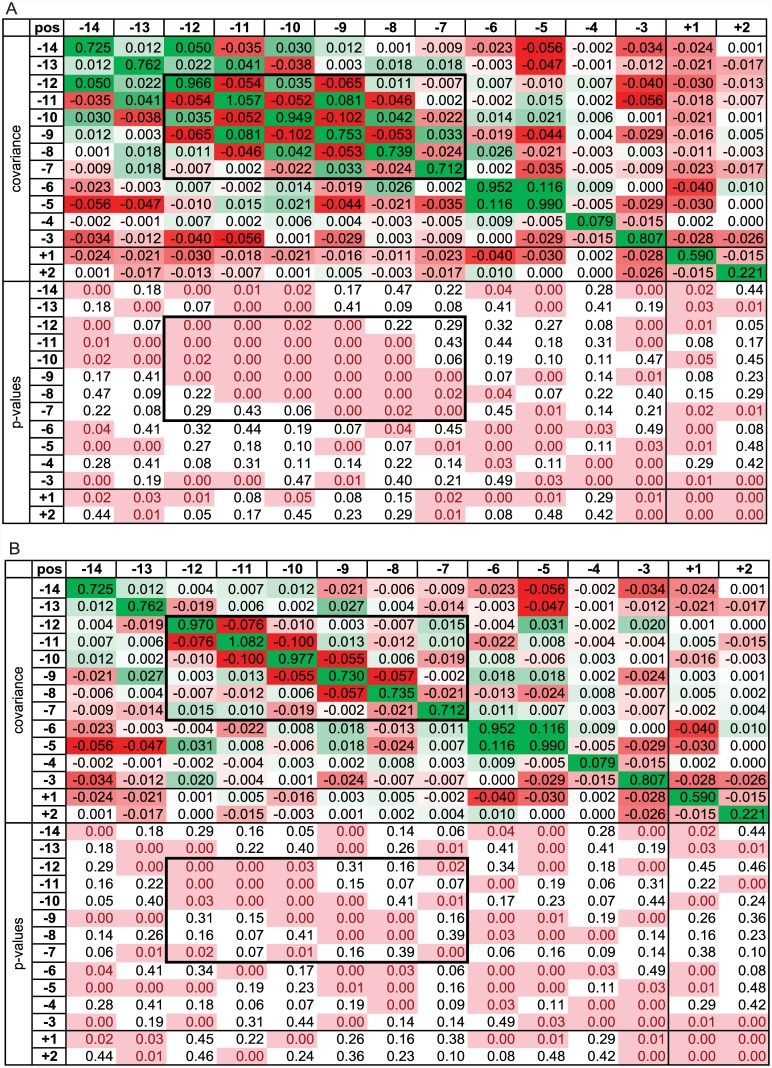
Covariance matrix of strength vectors for constitutive-exon ASSs (*M*. *musculus*). Notations are the same as in Fig 2. Positions (-12, -7) demonstrating the characteristic periodic pattern are marked by the black rectangle. (A) Original DSS sequences. (B) Artificial DSS sequences with the same dinucleotide content as the original sequences in positions (-12, -7) (see main text).

Probability that a given nucleotide occupies a particular position of a SS may depend on nucleotides occupying its other positions. Here, we reformulate this in terms of position strengths: does the strength of one position depend on the strength of another position? To answer this question, we constructed covariance matrices of strength vectors for donor and acceptor sites, with vector elements corresponding to strengths of each SS position. The covariance matrices of mouse DSSs and ASSs are shown in Figs 2 and 3. Matrices for the human and dog SSs are very similar (see S6 Table for DSSs and S7 Table for ASSs). The strength of each position within the exonic part of DSSs is negatively correlated with the strength of each position within the intronic part (here and below, the invariant GT and AG dinucleotides are not considered). This means that a high strength of the exonic part of DSS tends to be accompanied by a low strength of its intronic part and vice versa. In contrast, positions within the exonic part of the DSSs are positively correlated with each other. The intronic DSS positions +4(A), +5(G) and +6(T) are also positively correlated with each other; however, there is a negative correlation between position +3 and other intronic positions. The patterns in cassette-exon DSSs are similar, although the negative exon-intron correlations are more pronounced than in constitutive-exon DSSs (S6 Table).

Conceivably, a negative correlation between the exonic and the intronic parts of DSSs and positive correlations within the intronic and the exonic parts may result from signal migration from the exonic to the intronic part of SS [28]. To illustrate this, assume that the exonic parts were born perfect and deteriorate slowly, while intronic parts increase their strengths with time. Assume also that our sample contain DSSs of different ages. Young DSSs might have strong exonic parts and weak intronic parts, while old DSSs have the opposite characteristics. Pooling together DSSs of different ages may result in the observed correlations. To test this explanation, we subdivided our sample of DSSs into two subsamples (young and old, see Materials and Methods) and constructed covariance matrices of strength vectors for each subsample (S2 Fig). Matrices for young and old DSSs look very similar to those of the pooled sample (Fig 2), arguing against the above evolutionary history-based explanation. Therefore, although signal migration does contribute to the SS position strength changes in the course of evolution (see below), the observed pattern of negative and positive correlations cannot be explained by it, and is probably reflective of actual interactions (epistasis) rather than of mixing sites of different ages.

In the ASSs, the situation is more complicated (Fig 3). Both positive and negative correlations are abundant. In the PPT (positions -12 to -7), a characteristic periodic pattern is observed: strengths of adjacent positions (at distance of 1 nucleotide) are negatively correlated, strengths of positions at distance of 2 nucleotides are positively correlated, etc. (Fig 3A). A very similar pattern is observed in the human and dog branches, and at cassette-exon sites (S7 Table).

We hypothesized that the observed periodic pattern of correlations is caused by a biased dinucleotide content of the PPT. To test this hypothesis, we generated artificial sequences with the same dinucleotide content as in original sequences using a first-order Markov chain (see Materials and Methods). We then compared the covariance matrices of these artificial sequences with the covariance matrices of real ASS sequences (Fig 3B). The periodic pattern was also observed in the simulated data, although the covariations were slightly weaker. Therefore, the observed pattern is mainly determined by the dinucleotide content, with only a small contribution of more distant dependencies. More remote correlations also observed in the actual and, to a lesser extent, simulated data (Fig 3) largely arise from propagation of dinucleotide correlations for longer distances.

**Fig 4:**
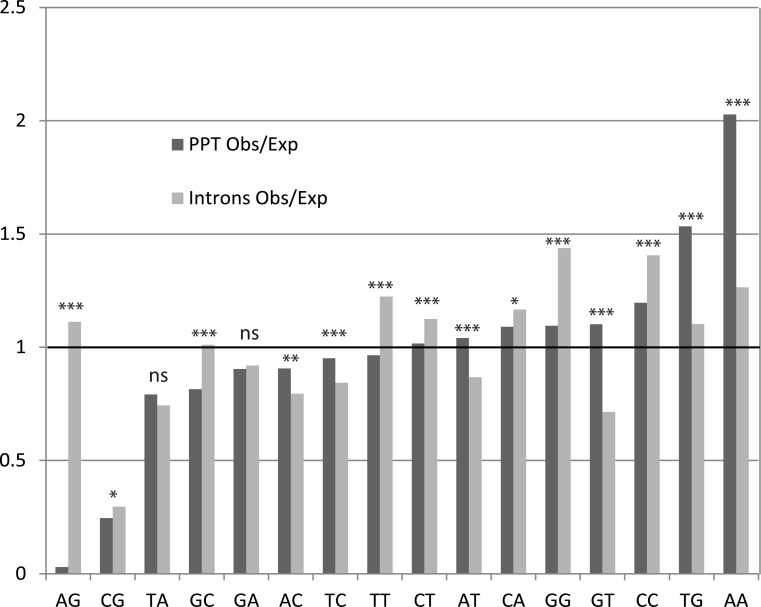
Dinucleotide frequencies within the region of periodic correlations (positions (-12, -7)) of PPT. Observed/expected ratio of dinucleotide frequencies within PPT (positions (-12, -7)) and within adjacent intron. Dinucleotides are ordered by the observed/expected ratio within PPT, from low to high. The expected values were obtained based on mononucleotide content. The significance of the difference between observed/expected ratio within PPTs and within adjacent introns are indicated: * 0.01 < p ≤ 0.05; ** 0.001 < p ≤ 0.01; *** p ≤ 0.001; ns p > 0.05.

To understand this pattern better, we ranked the dinucleotides at positions -12…-7 of ASSs by the degree of their under- or overrepresentation, compared with the expectations based on the mononucleotide content. We also estimated analogous values for the control region (adjacent intron). The most underrepresented dinucleotide was AG, which was ~30 times less frequent than expected in the ASSs, but not in the control regions (Fig 4). The AG dinucleotide is strongly selected against within PPT, likely due to avoidance of aberrant splicing [44] which probably at least partially explains the observed covariations (see Discussion). Existence of an intronic region depleted in AG was previously described as the AG exclusion zone (AGEZ) [45]. The second most underrepresented dinucleotide is CG, which was depleted by a factor of ~4, compared to the expectation if positions were independent. Unlike AG, CG was also underrepresented in introns (Fig 4). Its underrepresentation reflects the well-known phenomenon of CpG depletion in mammalian genomes [46] caused by the increased probability of methylated CpG to TpG mutation [47]. PPT dinucleotide content may be affected by wide range of factors including binding sites of U2AF65 and splicing regulatory proteins.

The long-distance pattern of alternating positive and negative correlations (Fig 3A) could also arise due to long tracts of identical dinucleotides, which may arise from replication slippage. However, the lengths of nearly all 16 periodic dinucleotides (AA)_n_, (AC)_n_,…(TT)_n_ were similar between simulated and real ASSs. The two exceptions were (TC)_n_ and (CT)_n_ (see S1 Fig), which tended to be slightly longer than expected.

In summary, the correlations in strengths of adjacent nucleotide positions may arise from (i) epistasis between positions within a SS, or from non-epistatic effects: (ii) pooling together of SSs at different stages of their evolution, (iii) non-selective mutational effects such as CpG dinucleotide avoidance, or (iv) selective effects that are not associated with site strength per se, such as ApG dinucleotide avoidance in ASSs. The covariances observed in ASSs likely arise mainly from (iv), while the covariances observed in DSSs likely reflect true epistasis (i).

### Strength changes at individual site positions in the course of evolution

Nucleotide substitutions at SSs can lead to changes in the strengths of corresponding positions. We can study how the mean strengths (averaged over all SSs of a particular type) of individual SS positions change in the course of evolution along specific phylogenetic branches.

For each SS position, the observed mean strength change *ΔŴ*_*O*_ *(i)*, i.e. the mean difference between the observed descendant and the reconstructed ancestral strengths, was compared to its expected value *ΔŴ*_*E*_ *(i)*, defined as the strength change that would have been obtained if the position has been evolving neutrally (see Materials and Methods). As expected, *ΔŴ*_*E*_ *(i)* is negative for all positions of DSSs and ASSs (S2 and S3 Tables), implying that if SSs evolved neutrally, they would diverge from the consensus. Obviously, the observed evolution of SSs is not neutral. Still, positions of SSs may evolve towards the consensus, leading to strength increase, or away from it, leading to strength decrease. To compare strength dynamics across positions and samples, we introduced the relative strength change $\omega =\frac{\Delta {\hat{W}}_{E}-\Delta {\hat{W}}_{O}}{\Delta {\hat{W}}_{E}}$. *ω* = 1 if the position remains unchanged, and *ω = 0* if it evolves neutrally. Since *ΔŴ*_*E*_ *(i) < 0*, *ω >1* when the strength increases, and *ω < 1* when strength decreases.

The values of *ω* for the DSS and ASS positions are shown in Tables 1 and 2, respectively. In all DSS positions, and in the vast majority of ASS positions, *ω>0*, implying that position strength is preserved by selection. Nevertheless, at many SS positions, strengths change significantly in one or more of the phylogenetic branches. This dynamics follows a complex pattern. Weakening positions numerically prevail; there are even positions where strength decreases more radically than expected neutrally, e.g. position –4 of the ASS. Nevertheless, there are many branches and positions in which the strength increased. Specifically, the strength tended to increase in intronic positions of constitutive DSSs, while it tended to decrease in the exonic positions.

**Table 1:** Relative strength changes ω for different positions of DSSs, in their evolution between the dog-human-mouse common ancestor and each of these species.

Position	Constitutive exons			Cassette exons		
	Dog	human	mouse	dog	human	mouse
**-3**	0.58	0.91	1.06	0.22	10.78	1.46
**-2**	**0.80**	**0.47**	**0.67**	1.00	1.00	0.99
**-1**	1.05	0.97	0.92	1.20	0.94	1.17
**3**	1.00	**1.05**	1.01	0.99	1.04	**0.95**
**4**	0.99	**1.06**	1.00	1.01	**0.82**	0.99
**5**	0.99	1.00	**1.04**	1.05	1.02	**1.09**
**6**	1.10	1.03	0.98	**1.23**	**0.66**	**0.79**

ω > 1 corresponds to increasing position strength, and ω < 1, to decreasing strength (see text). Cells are marked according to the p-value for strength change (i.e., deviation from ω = 1): boldface, p < 0.1; underlined, p < 0.05.

**Table 2:** Relative strength changes ω for different positions of ASSs, in their evolution between the dog-human-mouse common ancestor and each of these species.

Position	Constitutive exons			Cassette exons		
	dog	human	mouse	dog	human	mouse
-13	1.14	0.95	0.97	**0.25**	1.03	**0.74**
-12	**0.75**	**0.67**	0.92	**0.59**	0.55	**0.71**
-11	**0.84**	0.97	0.94	0.91	**0.61**	1.15
-10	0.92	0.92	1.04	1.11	1.04	0.76
-9	1.01	0.87	1.02	0.90	0.66	1.01
-8	**0.85**	1.08	1.05	0.91	**0.39**	1.22
-7	1.01	1.04	0.98	0.62	**0.21**	1.15
-6	0.95	**1.16**	1.00	0.97	**0.45**	1.12
-5	0.97	1.10	**1.13**	**0.78**	0.75	0.91
-4	**-0.40**	**-1.30**	**0.21**	-4.09	1.87	**-1.36**
-3	**0.96**	**0.95**	0.99	1.03	1.01	**1.07**
1	**0.96**	0.97	1.02	**0.90**	0.94	0.97
2	0.99	**1.50**	**0.83**	**-0.38**	2.22	0.81
3	-1.97	0.98	1.01	2.62	**-1.40**	**-1.76**

The notations are as in Table 1.

### Nucleotide substitutions at SS positions

In this section, we examine what nucleotide substitutions yield the strength changes observed in Tables 1 and 2. S4 Table shows the nucleotide substitutions that are significantly (p < 0.05) more or less frequent at a given position than expected neutrally (see S5 Table for the data on all nucleotide substitutions). The observed strength changes were due to a diverse pattern of positions-specific nucleotide frequency changes. Notably, the set of statistically significant nucleotide frequency changes does not explain all strength changes, and conversely, some significant nucleotide substitutions do not result in significant strength changes. Still, most of the strength changes are mainly driven by changing frequencies of just one or two nucleotides.

#### Donor splice sites

The decreasing strength of position -2 (A) of constitutive DSSs in the human and mouse branches (Table 1) is mainly due to the decreasing frequency of strong nucleotide A, and increasing frequency of the weakest nucleotide G (S4 Table). Weakening of position +4 in cassette-exon SSSs in the human branch is caused by a decreasing frequency of the strongest nucleotide A. The increase in frequency of strong nucleotide A, and decrease in frequency of weaker nucleotide G, at position +3 results in the increase of the strength of this position in the human branch, while the increase in frequency of A also results in the increased strength in the mouse branch. At position +5, the increase of strength in the mouse branch is caused by an increased frequency of the strongest nucleotide G.

#### Acceptor splice sites

Weakening of position -3 (C) in the mouse and dog branches in constitutive ASSs is caused by a decreasing frequency of the strongest nucleotide C, and an increasing frequency of a weaker nucleotide T. The opposite trend, strength growth associated with an increase in C frequency, is observed in the mouse branch for cassette exon ASSs. At position -4 (N), the frequencies of nucleotides are almost uniform, but this position demonstrates a large strength decrease (for sites of constitutive exons in the mouse and dog branches, and for cassette-exon sites, in the mouse branch). This may be explained by the fact that minor nucleotide preferences still exist at this position: the frequency of T (~30%) is higher than that of G (~20%), and the decrease of strength is caused mainly by increasing frequency of a weaker nucleotide G, and decreasing frequency of a stronger nucleotide T. The most pronounced trend in the PPT is a decrease in frequency of C and increase in the frequency of T; however, as T is only slightly stronger than C-, these changes do not lead to consistent strength changes.

In summary, in most cases, the strength changes are caused by changes of the frequency of the consensus nucleotide at a given position (e.g., nucleotide A in position -2 of DSS). In the PPT of ASS, substitutions between the two consensus nucleotides (C→T) were also observed.

### Are the dependences between positions preserved in the course of evolution?

Correlations between positions of extant SSs result from a long evolutionary history. In this study, we address the final stage of this evolution, from the common ancestor of human, mouse and dog to each of these species. Does the dependence between positions change the covariance matrices in the course of this evolution? In other words, do the SS substitutions that occur at different positions depend upon each other and, if yes, do they enhance or suppress the existing correlations? To address this, we compare the products of real evolution (where positions are not necessarily independent) with the products of simulated evolution (where positions within a SS evolve independently from each other). The average SS strengths were similar between the real and simulated data. In what follows, we compare the covariance matrices between the real and simulated data (S6 and S7 Tables). The deviation of the covariance matrices observed in the extant species from the simulated ones reflects what effect correlations between substitutions (e.g., due to functional epistatic interactions) have had on the currently observed correlations between positions.

In the DSSs, few significant differences between the covariance matrices were found, but some patterns emerged. Most importantly, the positive covariances within exonic parts and within intronic parts, and negative covariances between them, all tended to increase in magnitude. In addition, some DSS positions demonstrate branch-specific trends. Negative correlations between the exonic part of SS and position -1(G) increased in magnitude in the dog branch in constitutive-exon as well as cassette exon sites. At position +4(G), negative correlations with positions -1(G) and -2(A) increased in magnitude in the human branch.

In ASSs, the observed trends were again more mixed. The negative covariances within the intronic part of constitutive as well as cassette-exon sites were increasing in the mouse and human branches. On the contrary, in the exonic parts of ASSs, negative covariances were decreasing (in the human and mouse branches for constitutive-exon ASS, and in the human branch for cassette-exon sites). In the exonic parts of mouse cassette-exon ASSs, positive covariances were weakening. Negative covariances between the exonic and intronic parts were increasing in ASSs of constitutive exons (human, mouse), but not in cassette exons.

## Discussion

We showed that both the nucleotide content and the evolution of SS positions are non-independent. Before discussing the causes of the observed correlations, we address the possible artifacts that may lead to them.

### Sample preparation

Our results rely on correct inference of homologous functional SSs in different species. However, some SSs may be lost, gained, or otherwise experience a radical change in selection in some species since their divergence from the common ancestor. Admixture of SSs that are non-functional in some of the species can bias inferences of strength change.

To reduce this problem, we took care to include only SSs that are functional in all three species. Specifically, we required conservation of the key invariant dinucleotide (AG for DSSs, GT for ASSs). However, it may be possible that, at a deteriorating site, the invariant dinucleotide has not changed yet. In such a case, the genomic alignment alone would not be sufficient to detect the loss of functionality.

Functionality of a SS can be formally proven only by observation of the corresponding mRNA isoforms. However, requiring EST support from multiple species biases against relatively young sites, most of which are rarely used [40] and therefore are likely to be missed in some of the EST datasets, and especially against very young species-specific SSs [48]. Furthermore, in one of the species, the dog, the available EST data were rather limited: if we required EST support from this species, our sample size would be reduced by an order of magnitude. Therefore, we were limited to using EST support only from human and mouse; this is the approach used in the main text. To assess the robustness of this approach, we repeated the analysis using a ~4-fold larger sample in which only human, but not mouse, ESTs were taken into account. This had only a minor effect on the data quality statistics. Specifically, the ratio of the numbers of constitutive and cassette exons remained almost the same, as did the distribution of the inclusion rates (S1 Table). We observed a systematic bias in branch-specific strength changes: SS strengths decreased, on average, in the mouse and dog branches, and increased in the human branch, compared to the human-and-mouse-based sample (data not shown). This makes sense: as sites in the human-based sample were supported by ESTs only in the human genome, the mouse and dog site sets are potentially enriched by rarely used or even nonfunctional sites, introducing a bias towards apparent strength decrease in these species. In general, our results concerning patterns limited to just one of the phylogenetic branches should be treated with more caution than those concerning all three considered species.

Finally, even if the functionality of a SS is preserved, it could have changed its splicing type (constitutive to alternative or vice versa) between the considered species. However, the fraction of SSs that change their splicing type between human and mouse is low. When the mouse, rather than human, transcription data was used to classify the SSs into splicing types, the results were very similar (data not shown). Overall, our approach to inference of the strength change patterns is robust with respect to the organism used for splicing type classification.

### Transition matrix estimation

We compared the results obtained with our likelihood-based method of transition-matrix estimation (LB) with the standard parsimony-based method (PB). Generally, in all estimated transition matrices, the probability of a nucleotide to be unchanged is greater in PB than in LB. PB introduces a bias in estimation of observed strength changes: positions in the PB estimation substantially decreased their strengths from the ancestor to a descendant. No such effect is observed in the LB estimation (S8 Table).

### Dependence between positions and their evolution

Correlations between the nucleotide content of some exonic and intronic DSS positions have been described previously [35–37,49]. Here, we address such non-independencies in a systematic way, asking whether the strengths of positions within a SS are correlated with each other.

In DSSs, we observe that the strength of each position in the 5’ part of the site (spanning positions -3 to +3, i.e., including the entire exonic part and position +3 of the intronic part) is negatively correlated with the strength of each position within the 3’ part (spanning positions +4 to +6, i.e., including the remaining three positions of the intronic part). In contrast, the strengths of all positions in the 5’ part are positively correlated with each other, and the strengths of all positions in the 3’ part are also positively correlated with each other. Positive correlations within the intronic and exonic parts of SS, and negative correlations between them, could arise from pooling together SSs of different ages. However, these correlations are equally strong in separate subsamples of old and young SSs, demonstrating that SS ages are of minor importance at the considered time scale. As the correlations between nucleotides at opposite ends of a SS are as strong as those between adjacent nucleotides, they are also unlikely to be of mutational origin.

SS recognition by a spliceosome depends on several factors: presence of splicing enhancers or silencers [16], local transcription speed [50,51], chromatin structure [51–53], DNA methylation level [54] etc. ASS functionality also depends on branch point recognition by U2 [55]. Moreover, recognition of ASSs and DSSs are interdependent due to formation of cross-exon and cross-intron complexes [56].Strength of a SS position reflects not only degree of complementarity to U1 or ability to bind U2AF, but an integrated influence of all factors determining SS functionality.

One of the major factors influencing the DSS functionality is its complementarity to U1 snRNA. The consensus nucleotides are those that provide the maximal complementarity [8], and the strength of positions correlates well with the degree of complementarity. Indeed, the consensus sequence of DSS, 5’CAG|GURAGU3’, perfectly matches the corresponding sequence of U1: 3’GUCCAΨΨCA5’. Similarly, ASS functionality depends on its ability to bind U2AF. Stronger nucleotides are more conserved than weaker ones, implying that the differences in complementarity to U1 snRNA, or ability to bind U2AF, translate to differences in organismal fitness [27].

Epistasis is a phenomenon whereby fitness contribution of a particular allele located in one locus (position) depends on the allele in a second locus (position). In the absence of epistasis, fitness effects of two alleles are purely additive, i.e. the fitness contribution of these two alleles present simultaneously is the sum of the fitness contributions of individual alleles. In the case of epistasis, fitness effects of two alleles are not additive.

Thus, non-independence between strengths of different positions both within and between parts of SSs implies non-additivity of fitness effects of mutations, or epistasis. Positive correlations between strengths observed within SS parts imply that two strength-decreasing mutations together are less deleterious than expected from their effects when they occur alone (positive epistasis). Conversely, negative correlations between strengths observed between SS parts imply that two strength-decreasing mutations together are more deleterious than expected from their effects when they occur alone (negative epistasis).

We speculate that positive correlations in DSSs might be interpreted as cooperative binding of adjacent SS nucleotides with corresponding nucleotides of U1. Indeed, cooperative binding can take place due to stacking interactions between adjacent bases (we observe positive correlations in consecutive stretches of nucleotides within exonic and intronic parts). The nature of the negative interaction is more obscure. Possibly, there is some subpopulation of DSSs where maximum strength does not mean the highest fitness. For example, there is an experimental study showing that strengthening of weak SSs leads to loss of splicing [26]. These DSSs compensate for the low/high strength of the exonic part by the high/low strength of the intronic part, respectively. Previously, we have shown that the average selection on consensus nucleotides is lower for strong SSs, possibly reflecting the presence of a subpopulation of SSs mentioned above [27].

The existence of epistatic interactions between SS positions is supported by some experimental data, although the sign of the experimentally observed interactions is not necessarily consistent with our findings. A mispair with U1 at position +5 of DSS can be compensated by basepairing at positions +3 and +4 [57]. Experiments show that basepairing involving position -1 may rescue aberrant splicing caused by a mispair of position +6 with U1 in human cells [37], and that aberrant splicing caused by a mispair at position +5 is restored by pairing with the exonic part of DSS in *S*. *cerevisiae* [12], consistent with negative epistasis between mispairing mutations at different site pairs. Similarly, existence of a weaker nucleotide G at position +3 is only permitted when nucleotides at positions +4 to +6 form base pairs with U1 snRNA [58]; the same conclusion was reached by bioinformatic analysis [37]. The number of changes between human and mouse DSSs at the pairs of positions -1 and +5, and -2 and +5, was lower than expected if these positions had evolved independently, while at pairs of positions +4 and +5, and +5 and +6, it was higher than expected [37].

In ASSs, we observed that negative correlations of position strengths were frequent. Strengths of exonic position +1, +2 and intronic position -3 are negatively correlated with strengths of almost all other positions. Positions from -12 to -7 within the PPT demonstrate a periodic pattern of correlations: adjacent positions are negatively correlated, positions separated by one nucleotide are positively correlated, etc. This periodic pattern of correlations is caused mainly by the dinucleotide content, and in particular, by the depletion of the AG dinucleotide (AG exclusion zone, AGEZ).This depletion is likely caused by selection against spurious SSs which can disrupt normal splicing. Nucleotides A and G are weak in PPT; strong depletion of a dinucleotide consisting of two weak nucleotides increases the frequency of dinucleotides with two nucleotides of substantially different strengths (like TG), giving rise to the observed periodic pattern. AGEZ was shown to play an important role in splicing. In particular, experimental depletion of U2AF35 (which recognizes the AG dinucleotide) leads to up-regulation of some exons and down-regulation of others. ASSs of upregulated exons have longer AGEZs than downregulated or control exons [45].

Finally, we restored the SSs of the human-mouse-dog ancestor (with its own covariances between positions), and simulated its subsequent evolution in a process ignorant of these dependencies between positions. In DSSs, the covariances in the real data where stronger than those obtained in these simulations. This means that any dependencies between positions tended to increase, or at least to be retained, in the course of evolution of DSSs since the human-mouse-dog ancestor. Therefore, epistatic selection that has shaped these correlations in the first place is still in effect, and affects the patterns of substitutions. In the ASSs, the observed covariances differed from the simulated ones in different directions, suggesting that the selection pressure has been weak, or inconsistent in the course of evolution or between branches.

### Changes in SS position strengths and signal migration

Although we limited ourselves to studying SSs that were present in all three considered species, individual positions of SSs underwent changes in their strengths in some of the phylogenetic branches. While much of this dynamics had no apparent regularities, we still observed two specific patterns.

First, the strength of the exonic parts of constitutive DSSs tended to decrease, whereas the strength of the intronic part slightly increased (Table 1). A possible reason for this is signal migration from the exonic part to the intronic part of SS in the course of evolution. This phenomenon has been previously described for introns that were inserted before the divergence of major eukaryotic clades [28]. Here, we deal with much more recent timescales, and do not distinguish between very old and more recent introns, insofar as the intron origin predated the human-mouse-dog divergence. However, some introns appeared in the vertebrate branch after its divergence from insects [59], and some of our introns may still be relatively young, and experience ongoing signal migration. By contrast, we see no trace of signal migration in cassette-exon DSSs. One possible explanation for this is that cassette-exon SSs have a more complex system of regulation [9] which introduces additional constraints on strength and does not allow it to change substantially. We do not observe any evidence of signal migration in ASSs, which generally seem to undergo strength decrease both in the intronic and in the exonic parts. The latter result is consistent with the previous observation that the intronic parts of ASSs of different ages are not significantly different in strength [28].

Second, in the PPT of ASS, T is observed to accumulate at the expense of C. This suggests that the PPT is evolving to become more T-rich. Several experimental studies have shown that in general, the increasing number of T’s within the PPT facilitates splicing at the ASS [60,61]. However, isolated C’s in a T-rich PPT can support the use of an adjacent ASS [61,62], but several consecutive C’s can abolish detectable splicing [60,62]. Recent structural investigations of U2AF65 interaction with PPT clarify the picture. U2AF65 includes two RNA-recognition motifs which bind PPT: RRM1 and RRM2. RRM2 interacts with the 5’ region binding site within PPT and strongly prefers thymines. RRM1 which interacts with the 3’ part of binding site is more promiscuous: it allows C’s as well as T’s. Conformational changes of U2AF65 can also help to bind PPTs with different nucleotide content [63]. As a result, the balance of several selection pressures leads to accumulation of T’s within PPT.

In summary, by systematically addressing the patterns of covariances between SS nucleotides, we revealed that SSs experience a complex pattern of selective constraints. Both DSSs and ASSs show interdependencies between positions in terms of strengths. In particular, strengths of positions within exonic and within intronic parts of DSSs are positively correlated, and between exonic and intronic parts, negatively correlated; while ASSs show a characteristic periodic pattern of correlations. However, the reasons of these dependencies differ between DSSs and ASSs. The positive (negative) correlations between position strengths within DSSs are mainly caused by positive (respectively, negative) epistasis between corresponding substitutions. Consistently, selection shaped the pattern of substitutions in the course of relatively recent evolution of DSSs, so that positive covariances within the exonic and intronic parts, and negative covariances between them, all tended to increase in magnitude. In contrast, the main selective pressure leading to correlations between nucleotide content of different positions in ASSs was avoidance of the AG dinucleotide, possibly aimed at suppression of aberrant splicing. This selection against AG caused the characteristic periodic pattern of correlations in nucleotide strengths within the PPT of ASSs. Constitutive DSSs show a signature of signal migration: in the course of evolution, the average strength of the exonic part decreased, and the average strength of the intronic part either increased or remained the same. The ASSs showed no signs of signal migration. Finally, the PPTs had a tendency to become more T-rich due to an excess of the C-to-T substitutions. While our study revealed a high prevalence of epistatic interactions between SS positions, the exact functional basis of many of these interactions is unclear, and merits further research.

## Supporting Information

S1 Dataset Dataset of DSSs and ASSs used in this study. File names correspond to type of SS (DSS or ASS) and type of splicing (constitutive or cassette exons). Column names: id, internal SS id; hg19_chr_num, chromosome number (hg19 human genome annotation); hg19_chr_chain, chain; hg19_coord, coordinate of SS on corresponding chromosome; age, age of SS in K_s_ units; human_seq, sequence of SS in human genome (including the canonical dinucleotide of the SS, 18 adjacent intronic nucleotides, and 10 adjacent exonic nucleotides); mouse_seq, sequence of orthologous SS in mouse genome; dog_seq, sequence of orthologous SS in dog genome.
(ZIP)

S1 Fig Polynucleotide frequencies distribution. Red line, observed; blue line, expected. X axis, length of polynucleotide, Y axis, number of corresponding polinucleotides (log scale). (A) polyA; (B) poly(AC); (C) poly(AG), …, (P) polyT.
(EPS)

S2 Fig Covariance matrices of strength vectors for young and old SSs (constitutive-exon DSSs, *M*. *musculus*). Designations are the same as in Fig 2. (A) old DSSs (B) young DSSs.
(EPS)

S1 Table Distribution of exon inclusion frequencies before and after filtering. Number of sites and % of total cassette-exon sites are shown for each bin.
(XLSX)

S2 Table Expected strength changes *ΔŴ*_*E*_ in DSSs, for each position, branch (human, mouse and dog), and splicing type (constitutive and cassette exons). Position strengths were calculated based on the descendant sites as well as the ancestral sites. Negative values of Δ*Ŵ*_*E*_ are shown in red, with the color depth depending on the value. The corresponding p-values for the differences of the values from zero are also shown.
(XLSX)

S3 Table Expected strength changes *ΔŴ*_*E*_ in ASSs, for each position, branch (human, mouse and dog), and splicing type (constitutive and cassette exons). The notation is as in S2 Table.
(XLSX)

S4 Table Changes of frequencies of individual nucleotides in different positions of DSSs and ASSs. For each SS position, phylogenetic branch and splicing type, the nucleotides that significantly (p < 0.05) change their frequencies between the ancestor and the descendant are shown. “+” means increased frequency from the ancestor to the descendant, and “–” means decreased frequency. Cells with significant strength change, compared to the expected, are colored according to the direction of the change and the corresponding p-value: red, strength decrease (ω<1), p ≤ 0.05; orange, strength decrease (ω<1), 0.05 < p ≤ 0.1; green, strength increase (ω>1), p ≤ 0.05; chlorine, strength increase (ω>1), 0.05 < p ≤ 0.1.
(XLSX)

S5 Table Comparison of observed and expected nucleotide transition matrices. Columns, ancestor nucleotides; rows, descendant nucleotides. Observed (***P***_***O***_) and expected (***P***_***E***_) matrices, as well as the difference between them ***P***_***O***_—***P***_***E***_, are shown. Positive values of ***P***_***O***_—***P***_***E***_ are shown in green, while negative ones are shown in red (color depth depends on the absolute value). The significance of the difference between ***P***_***O***_(*i*)—***P***_***E***_ (*i*) and zero are shown; (p-values < 0.05 are marked as pink). Cases of positive selection are in **boldface and underlined**.
(XLSX)

S6 Table Comparison of covariance matrices of DSS strength vectors. Covariance matrices of strength vectors for real sites, artificial and the difference between these matrices (denoted as “real—artificial”) are shown. Positive elements of matrices marked as green, negative ones are shown in red (color depth depends on absolute value). Corresponding p-values that elements of covariance matrices (and of “real—artificial” matrix) differs from zero are represented (p-values < 0.05 marked as pink).
(XLSX)

S7 Table Comparison of covariance matrices of ASS strength vectors. Designations are the same as in S6 Table.
(XLSX)

S8 Table Comparison of observed strength changes calculated using LB and PB methods. Observed strength changes *ΔŴ*_*O*_ for the whole site, exonic and intronic parts of site are shown. Observed strength changes were calculated for different branches (human, mouse and dog) and splicing types (constitutive and cassette exons). LB and PB methods were considered separately. Statistical significance that *ΔŴ*_*O*_ differs from zero is also represented (p-values). Cases of strength decreasing (negative values) with p-value ≤ 0.05 are shown in red, ones with 0.05 < p-value ≤ 0.1 are shown in orange. Cases of strength increasing (positive values) with p-value ≤ 0.05 are shown in green, ones with 0.05 < p-value ≤ 0.1 are shown in chlorine.
(XLSX)

## References

[1] Collins L, Penny D. Complex Spliceosomal Organization Ancestral to Extant Eukaryotes. Mol Biol Evol. 2005;22: 1053–1066. 10.1093/molbev/msi091 15659557

[2] Sheth N, Roca X, Hastings ML, Roeder T, Krainer AR, Sachidanandam R. Comprehensive splice-site analysis using comparative genomics. Nucleic Acids Res. 2006;34: 3955–3967. 10.1093/nar/gkl556 16914448 PMC1557818

[3] Breathnach R, Chambon P. Organization and expression of eucaryotic split genes coding for proteins. Annu Rev Biochem. 1981;50: 349–383. 6791577 10.1146/annurev.bi.50.070181.002025

[4] Mercer TR, Clark MB, Andersen SB, Brunck ME, Haerty W, Crawford J, et al Genome-wide discovery of human splicing branchpoints. Genome Res. 2015; gr.182899.114. 10.1101/gr.182899.114 PMC4315302 25561518

[5] Taggart AJ, DeSimone AM, Shih JS, Filloux ME, Fairbrother WG. Large-scale mapping of branchpoints in human pre-mRNA transcripts in vivo. Nat Struct Mol Biol. 2012;19: 719–721. 10.1038/nsmb.2327 22705790 PMC3465671

[6] Michaud S, Reed R. An ATP-independent complex commits pre-mRNA to the mammalian spliceosome assembly pathway. Genes Dev. 1991;5: 2534–2546. 1836445 10.1101/gad.5.12b.2534

[7] Seraphin B, Rosbash M. Identification of functional U1 snRNA-pre-mRNA complexes committed to spliceosome assembly and splicing. Cell. 1989;59: 349–358. 2529976 10.1016/0092-8674(89)90296-1

[8] Roca X, Sachidanandam R, Krainer AR. Determinants of the inherent strength of human 5′ splice sites. RNA. 2005;11: 683–698. 10.1261/rna.2040605 15840817 PMC1370755

[9] Ast G. How did alternative splicing evolve? Nat Rev Genet. 2004;5: 773–782. 10.1038/nrg1451 15510168

[10] Kretzner L, Rymond BC, Rosbash M. S. cerevisiae U1 RNA is large and has limited primary sequence homology to metazoan U1 snRNA. Cell. 1987;50: 593–602. 2440584 10.1016/0092-8674(87)90032-8

[11] Schwartz S, Silva J, Burstein D, Pupko T, Eyras E, Ast G. Large-scale comparative analysis of splicing signals and their corresponding splicing factors in eukaryotes. Genome Res. 2008;18: 88–103. 10.1101/gr.6818908 18032728 PMC2134773

[12] Séraphin B, Kretzner L, Rosbash M. A U1 snRNA:pre-mRNA base pairing interaction is required early in yeast spliceosome assembly but does not uniquely define the 5’ cleavage site. EMBO J. 1988;7: 2533–2538. 3056718 10.1002/j.1460-2075.1988.tb03101.x PMC457124

[13] Zhuang Y, Weiner AM. A compensatory base change in U1 snRNA suppresses a 5’ splice site mutation. Cell. 1986;46: 827–835. 3757028 10.1016/0092-8674(86)90064-4

[14] Lerner MR, Boyle JA, Mount SM, Wolin SL, Steitz JA. Are snRNPs involved in splicing? Nature. 1980;283: 220–224. 7350545 10.1038/283220a0

[15] Segault V, Will CL, Polycarpou-Schwarz M, Mattaj IW, Branlant C, Luhrmann R. Conserved Loop I of U5 Small Nuclear RNA Is Dispensable for Both Catalytic Steps of Pre-mRNA Splicing in HeLa Nuclear Extracts. Mol Cell Biol. 1999;19: 2782–2790. 10082544 10.1128/mcb.19.4.2782 PMC84071

[16] Smith CWJ, Valcárcel J. Alternative pre-mRNA splicing: the logic of combinatorial control. Trends Biochem Sci. 2000;25: 381–388. 10.1016/S0968-0004(00)01604-2 10916158

[17] Manceau V, Swenson M, Le Caer J, Sobel A, Kielkopf CL, Maucuer A. Major phosphorylation of SF1 on adjacent Ser‐Pro motifs enhances interaction with U2AF65. FEBS J. 2006;273: 577–587. 10.1111/j.1742-4658.2005.05091.x 16420481 PMC1949809

[18] Valcárcel J, Gaur RK, Singh R, Green MR. Interaction of U2AF65 RS Region with Pre-mRNA of Branch Point and Promotion Base Pairing with U2 snRNA. Science. 1996;273: 1706–1709. 10.1126/science.273.5282.1706 8781232

[19] Shcherbakova I, Hoskins AA, Friedman LJ, Serebrov V, Corrêa IR, Xu M-Q, et al Alternative spliceosome assembly pathways revealed by single-molecule fluorescence microscopy. Cell Rep. 2013;5: 151–165. 10.1016/j.celrep.2013.08.026 24075986 PMC3927372

[20] Sorek R, Ast G. Intronic Sequences Flanking Alternatively Spliced Exons Are Conserved Between Human and Mouse. Genome Res. 2003;13: 1631–1637. 10.1101/gr.1208803 12840041 PMC403736

[21] Stamm S, Zhang MQ, Marr TG, Helfman DM. A sequence compilation and comparison of exons that are alternatively spliced in neurons. Nucleic Acids Res. 1994;22: 1515–1526. 10.1093/nar/22.9.1515 8202349 PMC308024

[22] Stamm S, Zhu J, Nakai K, Stoilov P, Stoss O, Zhang MQ. An alternative-exon database and its statistical analysis. DNA Cell Biol. 2000;19: 739–756. 11177572 10.1089/104454900750058107

[23] Clark F, Thanaraj TA. Categorization and characterization of transcript-confirmed constitutively and alternatively spliced introns and exons from human. Hum Mol Genet. 2002;11: 451 11854178 10.1093/hmg/11.4.451

[24] Baek D, Green P. Sequence conservation, relative isoform frequencies, and nonsense-mediated decay in evolutionarily conserved alternative splicing. Proc Natl Acad Sci U S A. 2005;102: 12813 16123126 10.1073/pnas.0506139102 PMC1192826

[25] Zheng CL, Fu X-D, Gribskov M. Characteristics and regulatory elements defining constitutive splicing and different modes of alternative splicing in human and mouse. RNA. 2005;11: 1777–1787. 10.1261/rna.2660805 16251388 PMC1370866

[26] Zheng Z-M, Quintero J, Reid ES, Gocke C, Baker CC. Optimization of a Weak 3’ Splice Site Counteracts the Function of a Bovine Papillomavirus Type 1 Exonic Splicing Suppressor In Vitro and In Vivo. J Virol. 2000;74: 5902–5910. 10.1128/JVI.74.13.5902-5910.2000 10846071 PMC112086

[27] Denisov SV, Bazykin GA, Sutormin R, Favorov AV, Mironov AA, Gelfand MS, et al Weak Negative and Positive Selection and the Drift Load at Splice Sites. Genome Biol Evol. 2014;6: 1437–1447. 10.1093/gbe/evu100 24966225 PMC4079205

[28] Sverdlov AV, Rogozin IB, Babenko VN, Koonin EV. Evidence of Splice Signal Migration from Exon to Intron during Intron Evolution. Curr Biol. 2003;13: 2170–2174. 10.1016/j.cub.2003.12.003 14680632

[29] Rogozin IB, Carmel L, Csuros M, Koonin EV. Origin and evolution of spliceosomal introns. Biol Direct. 2012;7: 1–28. 10.1186/1745-6150-7-11 22507701 PMC3488318

[30] Dibb NJ, Newman AJ. Evidence that introns arose at proto-splice sites. EMBO J. 1989;8: 2015–2021. 2792080 10.1002/j.1460-2075.1989.tb03609.x PMC401080

[31] Sadusky T, Newman AJ, Dibb NJ. Exon junction sequences as cryptic splice sites: implications for intron origin. Curr Biol CB. 2004;14: 505–509. 10.1016/j.cub.2004.02.063 15043816

[32] Gelfand MS. Statistical analysis and prediction of the exonic structure of human genes. J Mol Evol. 1992;35: 239–252. 10.1007/BF00178600 1518091

[33] Sverdlov AV, Rogozin IB, Babenko VN, Koonin EV. Reconstruction of Ancestral Protosplice Sites. Curr Biol. 2004;14: 1505–1508. 10.1016/j.cub.2004.08.027 15324669

[34] Irimia M, Penny D, Roy SW. Coevolution of genomic intron number and splice sites. Trends Genet. 2007;23: 321–325. 10.1016/j.tig.2007.04.001 17442445

[35] Burge C, Karlin S. Prediction of complete gene structures in human genomic DNA. J Mol Biol. 1997;268: 78–94. 10.1006/jmbi.1997.0951 9149143

[36] Thanaraj TA, Robinson AJ. Prediction of exact boundaries of exons. Brief Bioinform. 2000;1: 343–356. 10.1093/bib/1.4.343 11465052

[37] Carmel I, Tal S, Vig I, Ast G. Comparative analysis detects dependencies among the 5′ splice-site positions. RNA. 2004;10: 828–840. 10.1261/rna.5196404 15100438 PMC1370573

[38] Nurtdinov RN, Neverov AD, Mal’ko DB, Kosmodem’yanskii IA, Ermakova EO, Ramenskii VE, et al EDAS—A database of alternatively spliced human genes. Biophysics. 2006;51: 523–526. 10.1134/S0006350906040026 16909834

[39] Nurtdinov R, Neverov A, Favorov A, Mironov A, Gelfand M. Conserved and species-specific alternative splicing in mammalian genomes. BMC Evol Biol. 2007;7: 249 10.1186/1471-2148-7-249 18154685 PMC2231371

[40] Nurtdinov RN, Mironov AA, Gelfand MS. Rodent-specific alternative exons are more frequent in rapidly evolving genes and in paralogs. BMC Evol Biol. 2009;9: 142–142. 10.1186/1471-2148-9-142 19558667 PMC2711938

[41] Prasad AB, Allard MW, Green ED. Confirming the Phylogeny of Mammals by Use of Large Comparative Sequence Data Sets. Mol Biol Evol. 2008;25: 1795–1808. 10.1093/molbev/msn104 18453548 PMC2515873

[42] Barry D, Hartigan JA. Statistical Analysis of Hominoid Molecular Evolution. Stat Sci. 1987;2: 191–207. 10.1214/ss/1177013353

[43] Efron B, Tibshirani RJ. An introduction to the bootstrap. Boca Raton, Florida: CRC Press; 1993.

[44] Gelfand MS. Statistical analysis of mammalian pre-mRNA splicing sites. Nucleic Acids Res. 1989;17: 6369–6382. 10.1093/nar/17.15.6369 2528123 PMC318283

[45] Kralovicova J, Knut M, Cross NCP, Vorechovsky I. Identification of U2AF(35)-dependent exons by RNA-Seq reveals a link between 3′ splice-site organization and activity of U2AF-related proteins. Nucleic Acids Res. 2015;43: 3747–3763. 10.1093/nar/gkv194 25779042 PMC4402522

[46] Rollins RA, Haghighi F, Edwards JR, Das R, Zhang MQ, Ju J, et al Large-scale structure of genomic methylation patterns. Genome Res. 2006;16: 157–163. 10.1101/gr.4362006 16365381 PMC1361710

[47] Scarano E, Iaccarino M, Grippo P, Parisi E. The heterogeneity of thymine methyl group origin in DNA pyrimidine isostichs of developing sea urchin embryos. Proc Natl Acad Sci U S A. 1967;57: 1394–1400. 5231746 10.1073/pnas.57.5.1394 PMC224485

[48] Artamonova II, Gelfand MS. Evolution of the Exon–Intron Structure and Alternative Splicing of the MAGE-A Family of Cancer/Testis Antigens. J Mol Evol. 2004;59: 620–631. 10.1007/s00239-004-2654-3 15693618

[49] Rogozin IB, Milanesi L. Analysis of donor splice sites in different eukaryotic organisms. J Mol Evol. 1997;45: 50–59. 10.1007/PL00006200 9211734

[50] Kornblihtt AR, Mata MDL, Fededa JP, Muñoz MJ, Nogués G. Multiple links between transcription and splicing. RNA. 2004;10: 1489–1498. 10.1261/rna.7100104 15383674 PMC1370635

[51] Naftelberg S, Schor IE, Ast G, Kornblihtt AR. Regulation of alternative splicing through coupling with transcription and chromatin structure. Annu Rev Biochem. 2015;84: 165–198. 10.1146/annurev-biochem-060614-034242 26034889

[52] Agirre E, Bellora N, Alló M, Pagès A, Bertucci P, Kornblihtt AR, et al A chromatin code for alternative splicing involving a putative association between CTCF and HP1α proteins. BMC Biol. 2015;13: 31 10.1186/s12915-015-0141-5 25934638 PMC4446157

[53] Gonzalez I, Munita R, Agirre E, Dittmer TA, Gysling K, Misteli T, et al A lncRNA regulates alternative splicing via establishment of a splicing-specific chromatin signature. Nat Struct Mol Biol. 2015;22: 370–376. 10.1038/nsmb.3005 25849144 PMC6322542

[54] Lev Maor G, Yearim A, Ast G. The alternative role of DNA methylation in splicing regulation. Trends Genet TIG. 2015;31: 274–280. 10.1016/j.tig.2015.03.002 25837375

[55] Zhuang Y, Weiner AM. A compensatory base change in human U2 snRNA can suppress a branch site mutation. Genes Dev. 1989;3: 1545–1552. 10.1101/gad.3.10.1545 2612904

[56] Ram O, Ast G. SR proteins: a foot on the exon before the transition from intron to exon definition. Trends Genet. 2007;23: 5–7. 10.1016/j.tig.2006.10.002 17070958

[57] Nelson KK, Green MR. Mechanism for cryptic splice site activation during pre-mRNA splicing. Proc Natl Acad Sci U S A. 1990;87: 6253–6257. 2143583 10.1073/pnas.87.16.6253 PMC54511

[58] Ohno K, Brengman JM, Felice KJ, Cornblath DR, Engel AG. Congenital end-plate acetylcholinesterase deficiency caused by a nonsense mutation and an A—>G splice-donor-site mutation at position +3 of the collagenlike-tail-subunit gene (COLQ): how does G at position +3 result in aberrant splicing? Am J Hum Genet. 1999;65: 635–644. 10.1086/302551 10441569 PMC1377969

[59] Rogozin IB, Wolf YI, Sorokin AV, Mirkin BG, Koonin EV. Remarkable Interkingdom Conservation of Intron Positions and Massive, Lineage-Specific Intron Loss and Gain in Eukaryotic Evolution. Curr Biol. 2003;13: 1512–1517. 10.1016/S0960-9822(03)00558-X 12956953

[60] Coolidge CJ, Seely RJ, Patton JG. Functional Analysis of the Polypyrimidine Tract in pre-mRNA Splicing. Nucleic Acids Res. 1997;25: 888–896. 10.1093/nar/25.4.888 9016643 PMC146492

[61] Bouck J, Litwin S, Skalka AM, Katz RA. In vivo selection for intronic splicing signals from a randomized pool. Nucleic Acids Res. 1998;26: 4516–4523. 10.1093/nar/26.19.4516 9742257 PMC147856

[62] Roscigno RF, Weiner M, Garcia-Blanco MA. A mutational analysis of the polypyrimidine tract of introns. Effects of sequence differences in pyrimidine tracts on splicing. J Biol Chem. 1993;268: 11222–11229. 8496178

[63] Jenkins JL, Agrawal AA, Gupta A, Green MR, Kielkopf CL. U2AF65 adapts to diverse pre-mRNA splice sites through conformational selection of specific and promiscuous RNA recognition motifs. Nucleic Acids Res. 2013;41: 3859–3873. 10.1093/nar/gkt046 23376934 PMC3616741

